# Harnessing Odorant Receptor Activation to Suppress Real Malodor

**DOI:** 10.3390/ijms26041566

**Published:** 2025-02-13

**Authors:** Reina Kanemaki, Kahori Kishigami, Mei Saito, Masafumi Yohda, Yosuke Fukutani

**Affiliations:** 1 Department of Biotechnology and Life Science, Tokyo University of Agriculture and Technology, Koganei 184-8588, Tokyo, Japan; kanemaki-re@st-c.co.jp (R.K.); mei.saito@yohda.net (M.S.); yohda@cc.tuat.ac.jp (M.Y.); 2 Research Section, R & D Division, S.T. Corporation, Shinjuku 161-0033, Tokyo, Japan; k-kishi@st-c.co.jp

**Keywords:** odorant receptor, volatile compound, malodor, urine, suppressor, masking

## Abstract

Mammals, including humans, sense smell by the responses of odorant receptors (ORs) to odor molecules. We have developed an effective method to identify novel antagonists capable of suppressing the pungent odor of cat urine by targeting specific ORs. Since odors are typically complex mixtures of multiple volatile compounds, olfactory perception can vary depending on the composition. We analyzed the response of ORs to cat urine odor using vapor stimulation assays to identify the responding ORs. Gas chromatography–mass spectrometry was then performed to identify compounds eliciting responses from these ORs. Trace-amine-associated receptor 5 (TAAR5) demonstrated a significant response associated with the odor intensity of cat urine, identifying trimethylamine as a major contributor to the strong odor. From hundreds of candidate compounds, we identified several novel antagonists that exhibited greater efficacy than a known TAAR5 antagonist. These compounds not only reduced the responses of TAAR5-expressing cells to cat urine odor but also significantly reduced odor intensity and improved sensory pleasantness in human tests. Our findings suggest that targeting ORs responsive to specific odors, without isolating their individual components, is a promising strategy for developing deodorizing agents against complex malodors like cat urine odor. This study emphasizes the value of using real odor mixtures to enhance our understanding of odor perception.

## 1. Introduction

There are various odors in our living environments. Recently, pet odors have become a significant problem in daily life. In particular, the smell of cat urine is strong. Thus, the development of deodorant for pets is progressing [1,2,3,4]. Nevertheless, conventional methods, as described in previous studies, are often ineffective in open or large spaces [5]. Recently, sensory deodorization technology has been reported, which reduces malodor perception by suppressing the response of odorant receptors (ORs) to odor-causing substances [6]. While typical molecular components responsible for malodors include chemicals containing sulfur atoms, amine groups, and aldehyde groups, in reality, odors are complex mixtures of various volatile substances. 

ORs can be broadly categorized into canonical ORs [7,8], trace-amine-associated receptors (TAARs) [9,10], vomeronasal receptors [11,12], and membrane-spanning 4A (MS4A) proteins [13]. Humans have approximately 400 canonical ORs, which belong to the G-protein-coupled receptor (GPCR) family and are characterized by seven transmembrane domains [7,8,14,15,16]. Canonical ORs are the primary sensor proteins for odor perception, selectively responding to various odor molecules [17,18,19]. TAARs, also a component of the GPCR family, primarily respond to amine compounds [9,10]. Humans have six functional TAARs, whereas mice have 15. TAARs are less diverse than canonical ORs. Each mature olfactory sensory neuron expresses only one type of OR [10,20,21]. The combination codes generated by the responses of canonical ORs and TAARs make odor perception [22,23]. Although odors are complex mixtures of various volatile substances, specific ORs play a key role in determining the perception of malodors. In particular, some ORs that respond to cat urine odors are believed to induce discomfort; therefore, inhibiting these receptors could suppress the perception of malodors.

Cat urine contains a protein unique to cats, cauxin. Cauxin is an enzyme that hydrolyzes 3-methylbutanol-cysteinylglycine to felinine. Felinine is slowly degraded to 3-mercapto-3-methyl-1-butanol, which causes the specific smell of cat urine [24,25]. However, cat urine odor is a complex mixture of several volatile organic compounds (VOCs) [26], and the VOC composition of cat urine changes over time [27]. Hence, screening for odor suppressors based on single compounds may overlook effective candidates constituting cat urine odor. Moreover, most ORs that probably respond to cat urine odor remain unidentified, and the number of potential receptor candidates is vast.

As mentioned earlier, cat urine is a mixture of various substances. Testing deodorization strategies using the mixed odor is preferable to that focusing on single compounds. The composition of cat urine differs between individual cats depending on their living environment or diet. Even urine from a single cat varies in odor intensity with the collection day. Our goal was to identify compounds that could effectively suppress human perception of cat urine malodor based on high-throughput OR-expressing cell analysis. At first, we identified ORs that respond to the headspace gas of real cat urine (hereafter referred to as “cat urine odor”). Then, we screened for antagonistic fragrances that could inhibit these receptors. 

## 2. Results and Discussions

### 2.1. Identification of Cat-Urine-Responsive ORs

**Figure 1:**
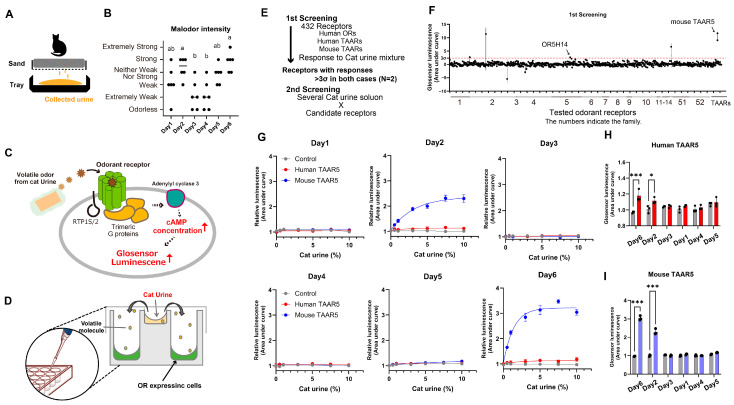
Identification of cat-urine-responsive odorant receptors. (**A**) Schematic of the method for collecting cat urine using a systematic toilet for cats. (**B**) Malodor intensity ratings of cat urine collected on different days as evaluated by six subjects. Nonparametric multiple comparisons were conducted using one-way analysis of variance (ANOVA) followed by Dunn’s multiple comparison test (*p* < 0.05). Different letters (a, ab, b) indicate significant differences between groups. (**C**) Schematic of the Glosensor assay showing the OR signal transduction pathway. Abbreviations: AC, adenylyl cyclase; ATP, adenosine triphosphate; cAMP, cyclic adenosine monophosphate; RTP1S/2, receptor transporting protein 1 short and 2. When odor molecules volatilized from the solution activate the ORs, the enzymatic reaction of Glosensor is triggered by the increase in cAMP concentration (red arrows). (**D**) Schematic of vapor stimulation using cat urine. (**E**) Screening strategy for OR selection. (**F**) Responses of selected human ORs, human TAARs, and mouse TAARs to the vapor phase from the cat urine mixture. The dotted line indicates the 3σ value calculated from all sample data. Each vertical line connects two scores for the same receptor, indicating a stable response. (**G**) Dose–response analysis of human and mouse TAAR5 to cat urine samples collected on different days. Human TAAR5 (red), mouse TAAR5 (blue), and vector control (gray). (**H**,**I**) Multiple comparisons for mouse TAAR5 (**H**) and human TAAR5 (**I**) responses were conducted using one-way analysis of variance (ANOVA) followed by Dunnett’s test (* *p* < 0.05, *** *p* < 0.001).

To identify the specific ORs responding to cat urine, we used the urine of a 7-year-old neutered male Scottish Fold cat, one of the most commonly maintained breeds in Japan [28]. Cat urine was collected from a tray under the layer of sand that does not absorb liquid in the system toilet box for more than 12 h and less than 24 h (Figure 1A). Evaluation of odor intensity of the collected urine showed that some samples had a strong, pungent odor, whereas others did not (Figure 1B). To minimize variability in the odor intensity and components, we used a mixture of urine samples collected on different days for the initial screening of ORs. The mixture was prepared with a ratio of 30% urine with a mild pungent odor and 5% urine with a strong pungent odor. The presence of a pungent odor was confirmed after adjusting the mixed solution.

For stimulating receptor-expressing cells, we added cat urine to the wells of assay plates containing Hana3A cells transfected with different ORs and then sealed the plates with lids to allow the volatilized urine odor to stimulate the cells within the plates (Figure 1C,D). In the initial screening, we identified receptors responsive to cat urine based on the threshold defined as the 3σ value (SD = 0.78, 3σ value = 2.35) calculated from the response data of all receptor-expressing cells (Figure 1E). This process resulted in the selection of mouse TAAR5 (average = 10.36) and human OR5H14 (average score = 2.35) (Figure 1F). Because mouse TAAR5 demonstrated a clearly higher response to cat urine than OR5H14, we performed a second screening to evaluate the concentration-dependent responses of the mouse TAAR5-expressing cells to varying dilutions of strong odor urine. Human TAAR5, the ortholog of mouse TAAR5, might not have reached the response threshold in the first screening due to differences in expression levels in Hana3A cells (Figure S1).

Human TAAR5 is known as a trimethylamine (TMA) receptor [10,29]. We further investigated the responses of both mouse and human TAAR5 to urine samples with varying pungent odors and found that both receptors responded robustly to the strongly pungent urine (Figure 1H,I). We also examined whether TAAR5-expressing cells responded to urine from cats of different types, sexes, and ages; TAAR5 showed a significant response to all tested samples (Figure S2). These findings suggest that the molecules acting as agonists for TAAR5 are responsible for the pungent odor of cat urine. 

Another OR that surpassed the 3σ value, OR10H2, responds to relatively pleasant odor components such as cinnamaldehyde and geranyl acetate as known agonists [23]. Because the purpose of this study was to suppress malodorous and irritating odors from cat urine, we first investigated whether the malodorous odor derived from cat urine could be suppressed by suppressing TAAR5. If the malodorous odor cannot be suppressed by TAAR5 inhibitors alone, it is considered preferable to treat these receptors as second or subsequent candidate targets.

### 2.2. Amine Compounds as Key Contributors in Cat Urine Odor

**Figure 2:**
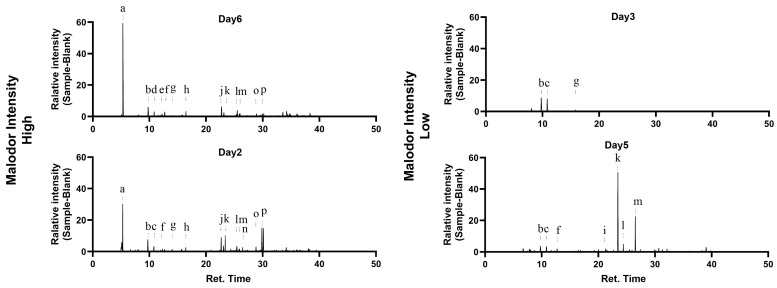
Identification of malodor-causing volatile compounds in cat urine.

The intensity of the pungent odor of cat urine varied depending on the collection date. Among the six urine samples, those from Day 2 and Day 6, which exhibited particularly strong odor and high TAAR5 response, were selected for analysis (Figure 1B,G). In contrast, urine samples from Day 3 and Day 5, which had weak odor and no TAAR5 response, were chosen as controls. To investigate the chemical basis of this variation, we analyzed multiple urine samples with different odor intensities by gas chromatography–mass spectrometry (GC-MS) after collecting volatile compounds from the headspace of cat urine using a solid-phase microextraction (SPME) fiber. Trimethylamine (TMA) was prominently detected in samples with a strong pungent odor (Peak a on Days 2 and 6) (Figure 2 and Table 1). TAAR5 responds to amines, including TMA, and it is predicted that amines are consistently present in pungent cat urine odors [26]. 

Identification occurred of sample-specific compounds in cat urine exhibiting strong malodor (Days 6 and 2) and weak malodor (Days 3 and 5) (refer to Figure 1B). Sample-specific peaks are labeled with letters. Refer to Table 1 for the corresponding compounds for each labeled peak.

In addition to TMA, we exclusively detected other compounds (Peaks d, e, j, o, and p on Days 2 and 6) in the pungent samples but none in the non-pungent samples (Days 3 and 5). Although these compounds lacked known associations with malodor, some were described as having a “nutty” aroma (referred to VCF online website, Version VCF16.10, released on 23 December 2022: https://www.vcf-online.nl/VcfHome.cfm, accessed on 8 October 2024), suggesting that they can influence the overall characteristic of cat urine odor, distinct from TMA. Remarkably, we did not detect previously reported odor compounds in cat urine, such as 3-mercapto-3-methylbutyl formate and 3-mercapto-3-methyl-1-butanol [24], in any of the samples. It is plausible that the levels of these compounds are less than the detection limit in neutered cats, where biosynthesis may be reduced.

Considering that TAAR5 demonstrated a significant response correlating with the intensity of the pungent odor, TMA may be a key fluctuating component responsible for producing this odor.

### 2.3. Identification of Effective Antagonists That Suppress Cat-Urine-Responsive Receptors

**Figure 3:**
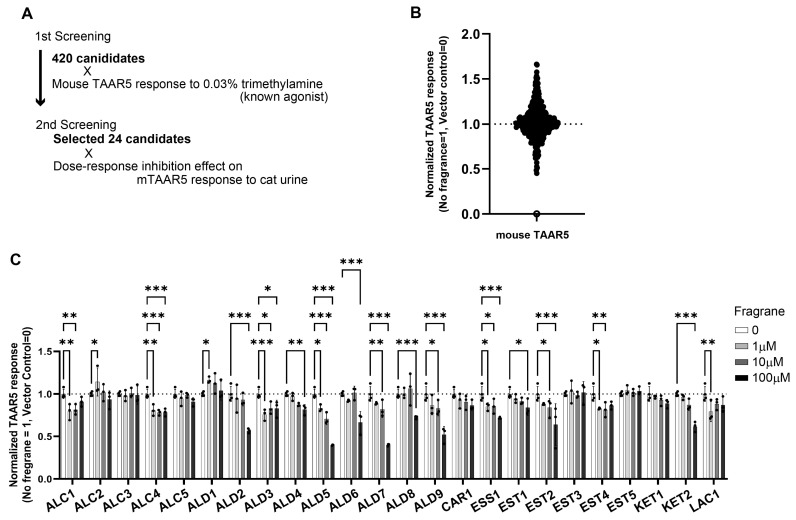
Antagonists of cat urine odorants responsive to TAAR5. (**A**) Outline of the antagonist screening procedure. (**B**) First screening of 400 odorants for the inhibition of TAAR5 responses to cat urine. (**C**) Dose-dependent inhibitory effect of candidate antagonists against TAAR5 in response to cat urine. Antagonist concentration: 0 (white), 1 µM (light gray), 10 µM (dark gray), and 100 µM (black). Error bars indicate standard deviation (s.d.), n = 3. Multiple comparisons were conducted using one-way analysis of variance (ANOVA) followed by Dunnett’s multiple comparison test (* *p* < 0.05, ** *p* < 0.01, *** *p* < 0.001). See Table S1 for details of each compound.

Not only mouse TAAR5 but also human TAAR5 responded to volatile compounds from cat urine (Figure 1H,I). Considering that human TAAR5 shares 86.6% sequence homology with mouse TAAR5 (Figure S1), we hypothesized that by screening for antagonists using mouse TAAR5, we could identify fragrances that also suppress human perception. Therefore, we screened for the antagonists of mouse TAAR5 to explore whether they could suppress cat urine odor. Since the amount of cat urine sample is limited, we used TMA-induced TAAR5 responses as a reference to screen for potential antagonists from a pool of 420 fragrance compounds (Figure 3A,B). 

In the primary screening of the 420 fragrance compounds, we selected the top 24 compounds with the strongest effects for a secondary screening using cat urine. The 24 candidate compounds were classified as follows: five alcohols (ALC), nine aldehydes (ALD), one carboxylic acid (CAR), one essential oil (ESS), five esters (EST), two ketones (KET), and one lactone (LAC) (Table S1). We identified several novel antagonists—ALC4 (santol pentenol), ALD2 (citral), ALD3 (muguet butanal), ALD5 (magnolia decadienal), ALD6 (trans-2-hexenal), ALD7 (cinnamaldehyde), ALD8 (floral butanal), ALD9 (helional), ESS1 (litsea cubeba fruit oil), and EST2 (ethyl safranate)—that inhibited TAAR5 responses to cat urine odor in a concentration-dependent manner. These antagonists outperformed the known TAAR5 antagonist timberol (1-(2,2,6-trimethylcyclohexyl)hexan-3-ol, designated ALC5 in this study) [29] (Figure 3C). Although there were relatively numerous molecules with aldehyde groups, we found no particular structural commonalities among the identified suppressors. Some of the antagonists also significantly suppressed the response of TAAR5 to TMA (Figure S3). Since TAAR5 exhibits high sensitivity to TMA, a higher concentration of inhibitor was expected to be required for suppression tests using pure TMA compared to the concentration of TMA present in cat urine. Among the identified compounds, those that demonstrated inhibitory effects against both cat urine and TMA were considered strong suppressor candidates.

### 2.4. Sensory Deodorization of Cat Urine Odor Using TAAR5 Antagonists

**Figure 4:**
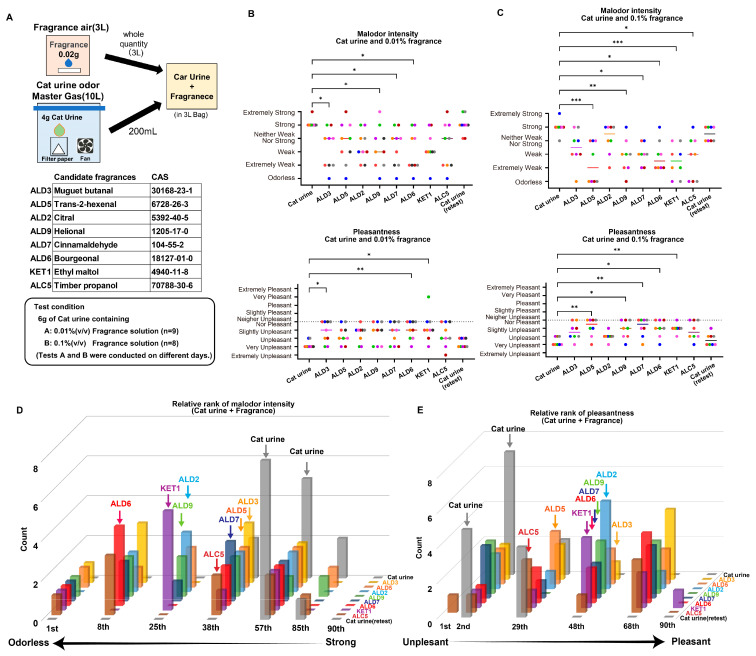
TAAR5 antagonists suppressed cat urine malodor. (**A**) Schematic of preparing gas mixture with cat urine and candidate fragrance compounds used in the human sensory evaluation test, candidate fragrance compounds used in this test, and the test condition. (**B**) Results of the evaluation test using 0.01% (*v*/*v*) fragrance solution showing the malodor intensity (top) and pleasantness (bottom). Each color represents the score of one subject. The median is shown as a black line in each condition. Nonparametric multiple comparisons against mean values were conducted using one-way analysis of variance (ANOVA) followed by Dunn’s multiple comparisons test (* *p* < 0.05, ** *p* < 0.01). (**C**) Results of the evaluation test using 0.1% (*v*/*v*) fragrance solution showing the malodor intensity (top) and pleasantness (bottom). Each color represents the score of one subject. The median is shown as a black line in each condition. Nonparametric multiple comparisons against mean values were conducted using one-way analysis of variance (ANOVA) followed by Dunn’s multiple comparisons test (* *p* < 0.05, ** *p* < 0.01, *** *p* < 0.001). (**D**,**E**) Relative rank in malodor intensity (**D**) and pleasantness (**E**). Median is shown as a color arrow in each condition.

The significance of this study is that the antagonists of the target odor receptors found in the in vitro evaluation system have been narrowed down to compounds that have been shown to suppress the perception of the odor in perception. We finally conducted a sensory deodorization test on eight fragrance compounds that demonstrated strong inhibitory effects in the cell assays. The eight odors were selected mainly from aldehydes, which had high TAAR5 response inhibition, and compounds with low TAAR5 inhibition, the previously reported antagonist ALC5 (also known as timberol). To prepare the fragrance solutions, we adjusted the concentration of each candidate fragrance compound (0.01% and 0.1%) to ensure that the odor intensity remained subtle. We placed 20 µL of each fragrance solution on a piece of filter paper inside a 3 L sampling bag filled with odorless air and allowed it to diffuse overnight (Figure 4A). For the cat urine odor, we created a master batch gas by volatilizing 4 g of cat urine in a 10 L sample bag. From this, we extracted 200 mL of gas and injected it into the fragrance air mixture to produce a combined gas sample with both cat urine and fragrance (Figure 4A). As a control, we used 3 L of odorless air mixed only with the cat urine odor. Nine participants evaluated the malodor intensity and pleasantness/unpleasantness of candidate fragrance compounds at 0.01% concentration, while eight participants evaluated them at 0.1% concentration, using a 6-point odor intensity scale and a 9-point hedonic scale, respectively (Figure 4A) [6]. 

Some fragrances selected based on the TAAR5 response significantly reduced malodor intensity and improved pleasantness derived from cat urine odor, outperforming the known TAAR5 antagonist timberol (ALC5), which was used as a reference (Figure 4B,C). Remarkably, even at the lower 0.01% concentration, ALD3 (also known as muguet butanal) and ALD6 (also known as bourgeonal) demonstrated significant suppression of both malodor intensity and unpleasantness (Figure 4B). Furthermore, ALD9 (also known as helional) and ALD7 (also known as cinnamaldehyde) significantly reduced malodor intensity, and ethyl maltol improved pleasantness. When a higher concentration of fragrance solution was used, more fragrance compounds exhibited odor-suppressing effects (Figure 4C). A relative ranking of odor-suppressing effects at the 0.01% concentration indicated that ALD6 (bourgeonal) exerted the highest malodor-suppressing effect (red in Figure 4D). Bourgeonal is a fragrance compound with a fresh, floral muguet scent and a watery green character and is commonly used in toiletries and alcoholic fragrances. ALD6 (bourgeonal) showed significant inhibition at relatively high concentrations compared to other aldehydes in the in vitro TAAR5 antagonist screening (Figure 3C and Figure S3). The inhibition of urinary odor in the sensory evaluation does not seem to necessarily correspond to the strength of the antagonist action of TAAR5. Our results suggest that bourgeonal effectively suppresses urine odor by acting as a TAAR5 antagonist. Remarkably, several human and mouse ORs respond to bourgeonal as an agonist, supporting that bourgeonal does not work as a universal inhibitor of odorant-responsive receptors [22,30]. This may be attributed to not only the deactivation of TAAR5 but also the activation of ORs that respond to bourgeonal, which probably resulted in a particularly effective alteration of the combination code, thereby improving the suppression of unpleasant odors. Additionally, four other fragrances, ALD8 (floral butanal), KET2 (alpha-damascone), EST2 (ethyl safranate), and ESS1 (litsea cubeba fruit oil), that inhibited TAAR5 response also demonstrated cat urine odor suppression in sensory evaluation (Figure S4).

The chemical structure of the fragrance summarizes the presence or absence of TAAR5 response inhibition effect on cat urine odor and its effect on sensory evaluation.

**Figure 5:**
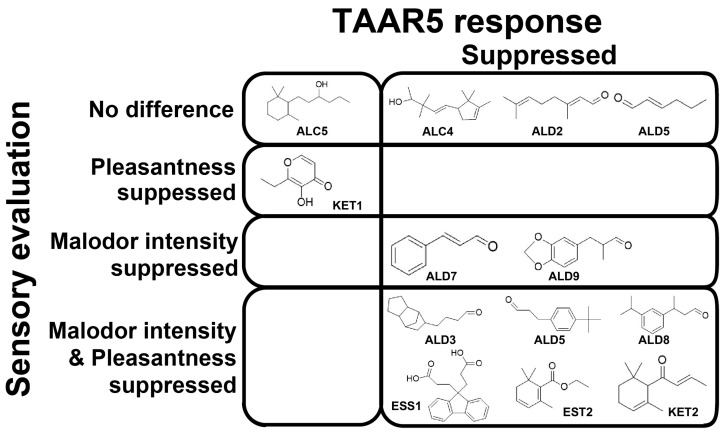
TAAR5 antagonists suppress cat urine malodor.

A comparison of TAAR5 response suppression and cat urine odor suppression in sensory evaluation is summarized in Figure 5. Among the eleven fragrances that suppressed TAAR5 response, eight also exhibited malodor suppression, with six of them significantly shifting pleasantness toward a more positive perception. Conversely three fragrances that suppressed TAAR5 response showed no positive effect in sensory evaluations. Furthermore, the two fragrances that did not suppress TAAR5 responses failed to reduce malodor intensity. These findings indicate that selecting masking fragrances based on olfactory receptor responses to odors is a highly effective approach. Moreover, predicting effective fragrances solely from molecular structures remains challenging, as no clear structural commonalities were identified among the most effective compounds.

As revealed by the GC-MS analysis, cat urine odor comprises multiple components, some of which act as agonists or antagonists for different receptors. It is challenging to predict molecules that exert antagonistic effects to positively influence the complex combination patterns of receptor activity. Of the hundreds of thousands of odor molecules, approximately 4000 are listed as fragrance compounds in The International Fragrance Association (IFRA) Transparency List that depicts the ingredients used by fragrance companies around the world (https://ifrafragrance.org/priorities/ingredients/ifra-transparency-list, accessed on 10 November 2024). However, it is impractical to investigate all these compounds solely through sensory tests. Therefore, a strategic approach that involves selecting specific ORs responsive to malodor sources and using receptor responses as indicators to narrow down effective antagonists is not only efficient but also reduces the exposure of sensory evaluators to malodors.

## 3. Materials and Methods

### 3.1. Cat Urine Collection

Urine was collected from a 7-year-old healthy and neutered male Scottish Fold cat. A litter box (Deo-Toilet Bin for small kitten to cat weighing ≤ 5 kg, Unicharm Corp, Ehime, Japan) was set overnight without an absorbent mat, and urine accumulated at the bottom of the litter box was collected using plastic pipettes in 50 mL glass vials. Cat urine voided from the previous afternoon or night until the following morning was collected and stored frozen. Because the presence of a pungent odor in the cat urine varied depending on the collection day, we used a mixture of non-pungent and pungent urine samples for the initial receptor screening, adjusting the concentration to a level that stimulated both a pungent odor and an unpleasant sensation. For experiments after the second screening, only the pungent urine samples were diluted and used. Urine from the different cat species, as shown in Figure S2, was collected by placing a camera in the same system toilet to record the cats during urination. The collected urine was filter-sterilized, sealed in 20 mL grass vials, and stored in a freezer.

### 3.2. Plasmid DNA Preparation

Open reading frames of human OR, human TAAR, and mouse TAAR genes were subcloned into the pCI vector (Promega, Madison, WI, USA) with an N-terminal Rho-tag (encoding the first 20 amino acids of rhodopsin). Plasmids for the expression of receptors, RTP1S [31,32], and pGlosensor F-22 (Promega) were amplified and purified using the Nucleospin plasmid TF-grade kit (Takara Bio, Shiga, Japan). All plasmid sequences were confirmed by Sanger sequencing performed by Eurofins Genomics Inc. (Tokyo, Japan).

### 3.3. Cell Culture

Hana3A [33] were maintained in Dulbecco’s modified Eagle’s medium (D-MEM) (FUJIFILM Wako Pure Chemical, Osaka, Japan) containing 10% FBS (*v*/*v*) (BioWest, Nuaillé, France) with L-alanyl-L-glutamine, penicillin–streptomycin, and amphotericin B at 37 °C and 5% CO_2_. No mycoplasma infection was detected.

### 3.4. Glosensor Assay

For detecting volatile cat urine odors, the Vapor Glosensor cAMP assay was used to evaluate changes in cAMP levels upon receptor activation by ligand binding [18]. Hana3A cells were plated on poly-l-lysine-coated 96-well plates. At 20–24 h after plating, the cells were transfected with 80 ng/well of plasmids encoding receptors, 5 ng/well of RTP1S [33], and 10 ng/well of Glosensor-22F plasmid (Promega) using the FuGene4K transfection reagent. After 20–24 h, the medium was replaced with 25 μL of HBSS(+) (FUJIFILM Wako Pure Chemical, Osaka, Japan) containing 10 mM HEPES and 1 mM glucose, followed by 25 μL of HBSS containing Glosensor cAMP Reagent (Promega). Then, the plates were incubated in the dark at 22 °C for 2 h to allow equilibrium with the reagent. Basal luminescence from each OR was measured before odor stimulation. After measuring the baseline luminescence, the cat urine solution was added either directly into each well or between the wells of the 96-well plate. Next, the plate was immediately covered and placed into the plate reader for measuring luminescence at 60 s intervals for 15 cycles. All luminescence values were normalized to the value obtained from cells transfected with the empty vector during the same cycle. The primary screening with all ORs corresponding to Figure 1F was performed in duplicate, and all subsequent experiments were performed in triplicate.

### 3.5. Screening for Antagonists 

Most of the compounds used were purchased from Merck (Darmstadt, Germany) Fujifilm wako pure chemical (Tokyo, Japan), Tokyo Chemical Industry Co. (Tokyo, Japan). Some were provided as samples by fragrance companies. All test compounds were prepared as stock solutions adjusted to 100 mM with DMSO. For primary screening of the antagonists, the initial testing involved adding each odorant as a solution to individual wells, followed by vapor stimulation with 0.03% (*v*/*v*) trimethylamine solution. At 1 day after transfecting TAAR5, RTP1S, and Glosensor plasmids, 25 μL of Glosensor solution was loaded for a 2 h incubation. Each odorant solution (600 μM) was prepared in HBSS buffer, and 5 μL of the 600 μM solution was added to the wells to a final concentration of 100 μM. After a 5 min luminescence measurement to evaluate the TAAR5 response to each odor, the cat urine solution was placed between the wells of the 96-well plate. The plate was then immediately covered and placed in the plate reader for measuring luminescence at 60 s intervals for 15 cycles. When evaluating the OR responses to each odorant, the luminescence values were normalized to the values obtained without the addition of fragrance compounds. For secondary screening by vapor stimulation with cat urine solution, each odorant solution (6, 60, and 600 μM (six times higher than the final concentration)) was prepared in HBSS buffer, and we added 5 μL each 6X solution to the wells at final concentrations of 1, 10, and 100 μM, respectively. The measurement method was the same as that used in the primary screening.

### 3.6. GC-MS Analysis

Cat urine samples stored at −18 °C were thawed at room temperature. A 1 mL aliquot of each sample was dispensed into a 50 mL glass vial. The headspace gas in the vial was replaced with pure air and sealed with a septum-equipped lid for the SPME fiber inlet. A Solid-Phase Microextraction (SPME) fiber (50/30 μm DVB/CAR/PDMS, Stable-flex 24Ga) was inserted through the septum and used to collect volatile compounds from the headspace for 30 min at room temperature. Volatile substances were analyzed using gas chromatography–mass spectrometry (GC-MS) (GCMS-QP2020, Shimadzu Corporation, Kyoto, Japan) equipped with an InterCap Pure-WAX column (60 m × 0.25 mm i.d., film thickness = 0.25 μm, GL Sciences Inc., Tokyo, Japan). The injector was set at 250 °C with the injector split ratio set to 10:1. The column temperature was held at 35 °C for 2 min, then increased to 240 °C at 5 °C/min, and finally held at 250 °C for 10 min. Helium was used as the carrier gas. The interface and ion source temperatures were maintained at 250 °C. The mass spectrometer was operated in electron impact mode at an electron energy of 70 eV and ion source temperature of 200 °C. Mass spectra were obtained in fullscan mode from m/z 40 to m/z 400. Compounds were identified by comparing their mass spectra to the National Institute of Standards and Technology (NIST) Mass Spectral Database and the Mass Spectra of Flavors and Fragrances of Natural and Synthetic Compounds (FFNSC). Data analysis was performed using GCMS Solution software (ver. 4.45) (Shimadzu Corp.).

### 3.7. Sensory Evaluation Test

The study protocol was approved by the Ethics Committee of ST Corporation, and written informed consent was obtained from all participants. We included male and female participants in their 20s and 30s. No analyses were stratified by age or sex. To prepare the fragrance solutions, the concentration of each candidate flavor compound was adjusted to final concentrations of 0.01% and 0.1% (*v*/*v*) with triethyl citrate, based on 10% solutions with DMSO as solvent, to ensure subtle aroma intensity. Standard odor paper (Daiichi Yakuhin Sangyo Co., Tokyo, Japan) was cut into 4 cm strips, impregnated with 0.02 g of the fragrance solution, and placed into a 3 L sampling bag filled with odorless pure air filtered through silica and activated charcoal. The sampling bag was left overnight at room temperature to allow the fragrance to diffuse. The gas was then transferred to a new 3 L sampling bag. For the cat urine odor, a master batch gas was created by volatilizing 4 g of cat urine in a 10 L sample bag. From this master batch, 200–300 mL of gas was extracted and mixed with the fragrance air to produce a combined gas sample containing both cat urine and the fragrance (Figure 4A). As a control, we used 3 L of odorless air mixed with only the cat urine odor. Participants (n = 8–10) evaluated the odor intensity and pleasantness/unpleasantness of the samples using a 6-point odor intensity scale and a 9-point hedonic scale, respectively (Figure 4A and Supplementary Figure S4). The index of odor intensity in the sensory evaluation test was evaluated using 5-point levels of strength of the odor strength standard solution adjusted with n-butanol (Japan Association on Odor Environment) [34]. The index of pleasantness was evaluated according to the 9-level pleasure and discomfort display method (set by the sensory test team in a survey on the establishment of odor standards commissioned by the Environment Agency (Japan Environmental Sanitation Center, 1971)). Panelists were divided into two groups, and half of the subjects reversed the evaluation order.

### 3.8. Statistical Analysis

Multiple comparisons were conducted using one-way analysis of variance (ANOVA) followed by Dunnett’s test, Tukey’s test, and Dunn’s test using GraphPad Prism. The average value is expressed as mean ± standard error. 

## 4. Conclusions

This study successfully identified fragrance compounds that suppress cellular responses and exert sensory deodorization effects in humans when tested with real cat urine. Analyzing the composition of odor components in ORs using intact odors would be highly effective. Notably, the most effective suppressors could not be identified solely through in vitro receptor assays. Since suppressor molecules themselves often possess odors, they can influence the response patterns of non-target ORs, making it challenging to predict changes in human odor perception based solely on cellular assays. Therefore, combining cellular assays with sensory evaluation tests is crucial for efficiently narrowing down candidates and identifying truly effective suppressors.

## 5. Patents

R.K., K.K., M.S., and Y.F. filed patent applications relevant to this work.

## Figures and Tables

**Table 1:** Cat-urine-specific compounds detected by GC-MS.

				Peak Area			
	Ret. Time (min)	Name	Odor Description	Day6 Strong Malodor	Day2 Strong Malodor	Day3 Weak Malodor	Day5 Weak Malodor
a	5.31	Trimethylamine	ammonia, fish, pungent, rotten	2.2 × 10^6^	7.1 × 10^5^	N/D	N/D
b	9.72	2-Pentanone	burnt plastic, ether, fruit, kerosine, ketone	4.5 × 10^5^	4.3 × 10^5^	7.3 × 10^5^	2.8 × 10^5^
c	10.82			N/D	1.0 × 10^5^	5.0 × 10^5^	2.4 × 10^5^
d	10.85	1,1,3,3,5,5,7,7-Octamethyl-7-(2-methylpropoxy)tetrasiloxan-1-ol		2.1 × 10^5^	N/D	N/D	N/D
e	12.72	2,3,3-Trimethyloctane		2.7 × 10^5^	N/D	N/D	N/D
f	13.87	gamma-decalactone	apricot, fat, fruit, peach, pleasant	2.4 × 10^5^	N/D	N/D	N/D
g	14.07	4-Heptanone	Fruit	2.6 × 10^5^	2.4 × 10^5^	1.0 × 10^5^	N/D
h	18.72	Hexyl acetate	apple, banana, confectionery, fruit, grass	2.0 × 10^5^	N/D	9.1 × 10^4^	1.2 × 10^5^
i	21.28	Silane, trimethyl[(1-methylethylidene)cyclopropyl]-		N/D	N/D	N/D	1.2 × 10^5^
j	23.15	2-Methyl-4-decanone		8.3 × 10^4^	1.4 × 10^5^	N/D	N/D
k	23.47	3-Methoxy-3-methylbutanol		8.5 × 10^5^	5.5 × 10^5^	5.4 × 10^5^	3.8 × 10^6^
l	25.32	Isovanillin, TBDMS derivative		7.1 × 10^4^	4.7 × 10^4^	N/D	N/D
m	25.52	Pyrrole	nut, sweet	2.3 × 10^5^	2.0 × 10^5^	N/D	1.1 × 10^5^
n	25.91	2-Hydroxy-iso-butyrophenone		N/D	1.2 × 10^5^	N/D	1.6 × 10^5^
o	28.88	5-Tridecanone	harsh, herb, oil, old nut, spice	9.2 × 10^4^	1.2 × 10^5^	N/D	N/D
p	30.59	Di-sec-Butyl ether		N/D	8.8 × 10^4^	N/D	N/D

## Data Availability

The original contributions presented in the study are included in the article/Supplementary Material; further inquiries can be directed to the corresponding author.

## Supplementary Materials

The following supporting information can be downloaded at https://www.mdpi.com/article/10.3390/ijms26041566/s1.
