# Supplementary material for: Harnessing Odorant Receptor Activation to Suppress Real Malodor
Source: Int J Mol Sci. 2025 Feb 13;26(4):1566. doi: 10.3390/ijms26041566 (PMC11855274; doi:10.3390/ijms26041566)
Supplement: Supplementary file 1 [file ijms-26-01566-s001.zip › ijms-3409698-supplementary.pdf]

Supplementary Information

Title

Harnessing Odorant Receptor Activation to Suppress Real Malodors

Authors

Reina Kanemaki[1,2], Kahori Kishigami[2], Mei Saito[1], Masafumi Yohda[1], Yosuke Fukutani[1]*

*Correspondence: fukutani@cc.tuat.ac.jp; Tel.: +81-42-388-7479

Affiliations

[1] Department of Biotechnology and Life Science, Tokyo University of Agriculture and Technology, Koganei, Tokyo 184-8588, Japan

[2] Research Section, R & D Division, S.T. Corporation, Shinjuku 161-0033, Tokyo, Japan

Table S1 Candidate compounds narrowed down by primary screening

	Name	CAS No	IUPAC	also known as
1	ALC1	28219-61-6	(2E)-2-ethyl-4-(2,2,3-trimethyl-3-cyclopenten-1-yl)-2-buten-1-ol	sandalrome,bacdanol
2	ALC2	67801-20-1	3-methyl-5-(2,2,3-trimethylcyclopent-3-en-1-yl)pent-4-en-2-ol	sandal pentenol, ebanol
3	ALC3	55066-48-3	3-methyl-5-phenylpentan-1-ol	rose absolute pentanol, mefrosol
4	ALC4	107898-54-4	(4E)-3,3-dimethyl-5-(2,2,3-trimethylcyclopent-3-en-1-yl)pent-4-en-2-ol	santol pentenol, polysantol
5	ALC5	70788-30-6	1-(2,2,6-trimethylcyclohexyl)hexan-3-ol	timber propanol, timberol
6	ALD1	112-44-7	undecanal	undecanal, hendecanal
7	ALD2	5392-40-5	3,7-dimethylocta-2,6-dienal	citral
8	ALD3	30168-23-1	4-(Octahydro-4,7-methano-5H-inden-5-ylidene)butanal	muguet butanal, dupical
9	ALD4	71077-31-1	4,8-Dimethyldeca-4,9-dienal	magnolia decadienal, floral super
10	ALD5	6728-26-3	(E)-hex-2-enal	(E)-2-hexenal, trans-2-hexenal
11	ALD6	18127-01-0	3-(4-tert-Butylphenyl)propanal	cyclamen propanal, bourgeonal
12	ALD7	104-55-2	(2E)-3-phenylprop-2-enal	cinnamaldehyde
13	ALD8	125109-85-5	3-[3-(propan-2-yl)phenyl]butanal	floral butanal, florhydral
14	ALD9	1205-17-0	3-(1,3-Benzodioxol-5-yl)-2-methylpropanal	ocean propanal, helional
15	CAR1	99-87-6	1-methyl-4-propan-2-ylbenzene	para-cymene, 4-methyl-1-isopropylbenzene
16	ESS1	68855-99-2	Litsea Cubeba Oil	litsea cubeba fruit oil
17	EST1	141-16-2	3,7-dimethyloct-6-enyl butanoate	citronellyl butyrate
18	EST2	35044-59-8	Ethyl 2, 6, 6-trimethylcyclohexa-2, 4-diene-1-carboxylate	ethyl safranate,
19	EST3	35836-72-7	(1R,5S)-2-(6,6-dimethylbicyclo[3.1.1]hept-2-en-2-yl) ethyl acetate	(1R)-(-)-nopyl acetate
20	EST4	151-05-3	(2-methyl-1-phenylpropan-2-yl) acetate	dimethyl benzyl carbinyl acetate
21	EST5	105-95-3	1,4-dioxacycloheptadecane-5,17-dione	ethylene brassylate
22	KET1	4940-11-8	2-Ethyl-3-hydroxy-4H-pyran-4-one	ethyl maltol
23	KET2	43052-87-5	(E)-1-(2,6,6-trimethylcyclohex-2-en-1-yl)but-2-en-1-one	alpha-damascone
24	LAC1	2305-05-7	5-octyloxolan-2-one	gamma-dodecalactone

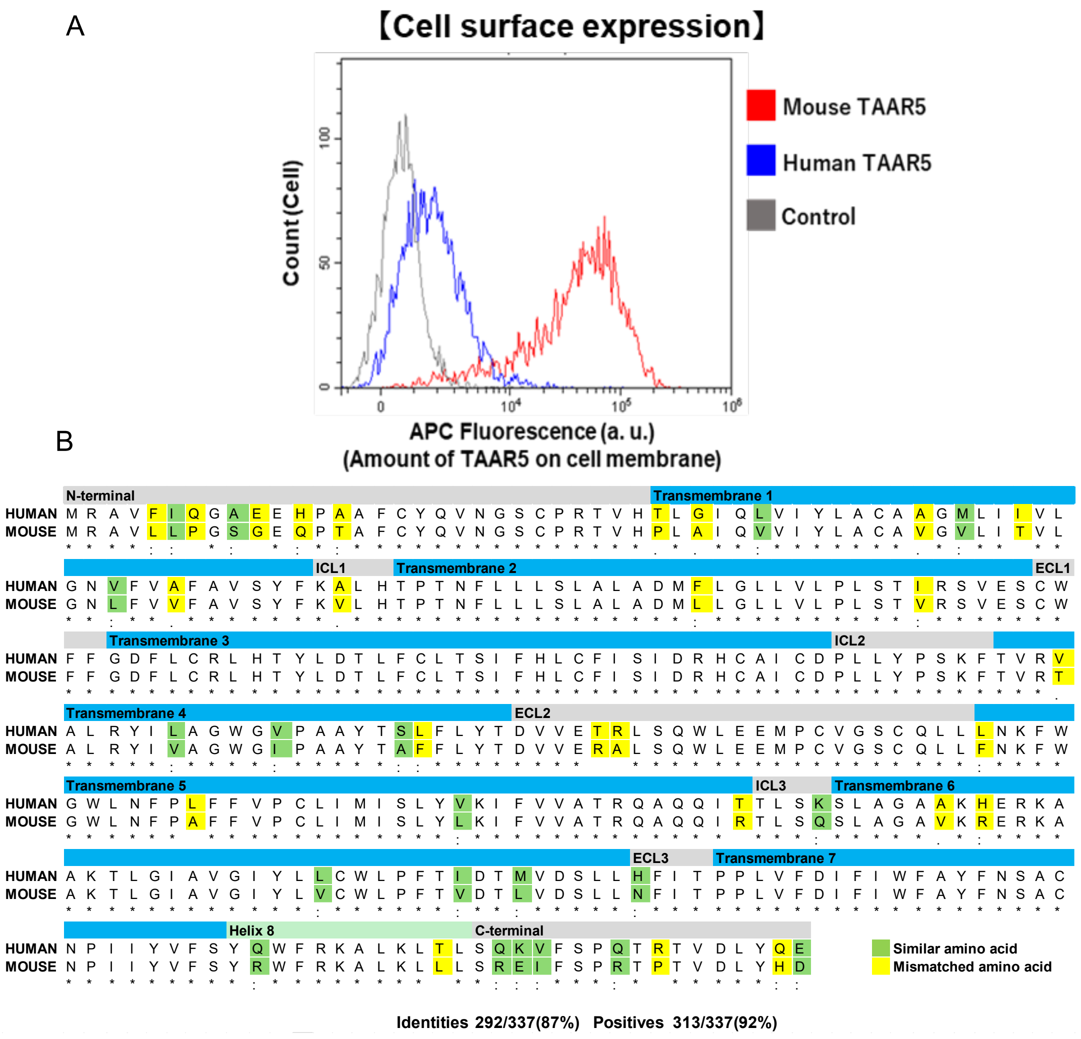

Figure S1: Differences in cell surface expression between mouse and human TAAR5

A: Cell surface expression of mouse TAAR5 (red) and human TAAR5 (blue) analyzed by flow cytometry.

B: Amino acid sequence alignment of mouse and human TAAR5.

A

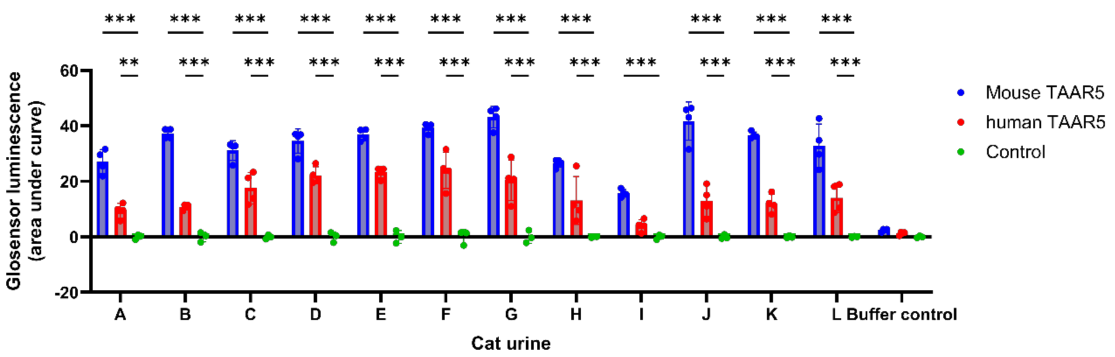

B

	Age	Sex	Castration	Breed
A	0	♀	not yet	Savannah cat
B	8	♂	neutered	MIX (Oriental x Himalayan)
C	12	♀	neutered	American Curl
D	3	♂	neutered	Scottish fold
E	1	♂	neutered	Ragdoll
F	5	♂	neutered	Maine Coon
G	8	♀	not yet	Bengal cat
H	1	♂	neutered	Bombay
I	1	♀	not yet	Munchkin
J	6	♀	neutered	American Shorthair
K	3	♂	neutered	Scottish fold
L	1	♀	not yet	Burmilla

Figure S2: Response of TAAR5-expressing cells to various cat urine samples

A: Collected cat urine was added into the TAAR5-expressing cells, and the response of TAAR5-expressing cells positioned between the wells was assessed using Glosensor luminescence. Data are presented as the area under the curve (AUC), normalized to the control sample. Error bars represent the standard deviation (s.d.), n = 4. Statistical significance was determined using one-way analysis of variance (ANOVA) followed by Dunnett's multiple comparison test (**$p < 0.01$, ***$p < 0.001$).
B: Information (Age, Sex, Castration and Breed) of each cat.

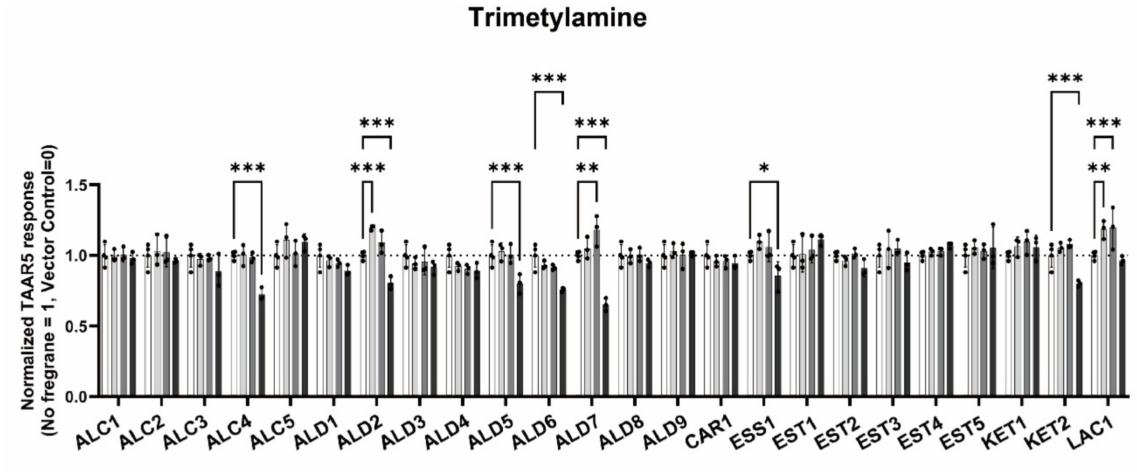

Figure S3: Dose-dependent inhibitory effect of candidate antagonists against TAAR5 in response to trimethylamine.

Antagonist concentration; 0 (white), 1 μM (light gray), 10 μM (dark gray), and 100 μM (black). Error bars indicate standard deviation (s.d.), n = 3. Multiple comparisons were conducted using one-way analysis of variance (ANOVA) followed by Dunnett's multiple comparison test (*p < 0.05, **p < 0.01, ***p < 0.001).

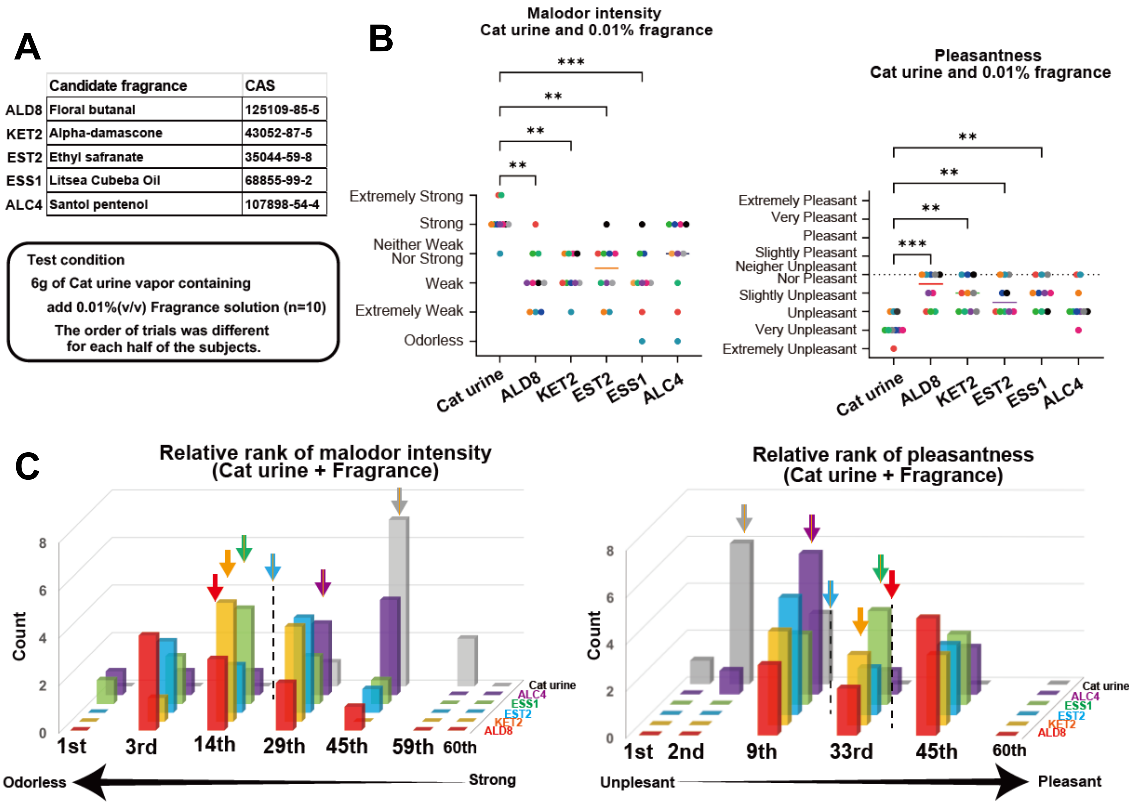

Figure S4. TAAR5 antagonists suppress cat urine malodor
(A) candidate fragrance compounds used in this test, and test condition. (B) Results of evaluation test using 0.01% (v/v) fragrance solution showing the malodor intensity (left) and pleasantness (right). Each color represents the score of one subject. The median is shown as a black line in each condition. Nonparametric multiple comparisons against mean values were conducted using one-way analysis of variance (ANOVA) followed by Dunn's multiple comparisons test (**p < 0.01, ***p < 0.001). (C) Relative rank in malodor intensity (left) and pleasantness (right). Median is shown as a color arrow in each condition.
